# Variable ventilation ages in the equatorial Indian Ocean thermocline during the LGM

**DOI:** 10.1038/s41598-023-38388-z

**Published:** 2023-07-13

**Authors:** J. Raddatz, E. Beisel, M. Butzin, A. Schröder-Ritzrau, C. Betzler, R. Friedrich, N. Frank

**Affiliations:** 1 Institute of Geosciences, Goethe University Frankfurt, Altenhöferallee 1, 60438 Frankfurt Am Main, Germany; 2 Institute of Environmental Physics, Heidelberg University, Im Neuenheimer Feld 229, 69120 Heidelberg, Germany; 3 MARUM-Center for Marine Environmental Sciences, University of Bremen, P.O. Box 330440, 28334 Bremen, Germany; 4 Center for Earth System Research and Sustainability, Institute of Geology, University of Hamburg, Bundesstraße 55, 20146 Hamburg, Germany; 5 Curt-Engelhorn-Center Archaeometry, C4, 8, 68159 Mannheim, Germany

**Keywords:** Biogeochemistry, Climate sciences, Ocean sciences

## Abstract

Variations of atmospheric CO_2_ during the Pleistocene ice-ages have been associated with changes in the drawdown of carbon into the deep-sea. Modelling studies suggest that about one third of the glacial carbon drawdown may not be associated to the deep ocean, but to the thermocline or intermediate ocean. However, the carbon storage capacity of thermocline waters is still poorly constrained. Here we present paired ^230^Th/U and ^14^C measurements on scleractinian cold-water corals retrieved from ~ 450 m water depth off the Maldives in the Indian Ocean. Based on these measurements we calculate ∆^14^C, ∆∆^14^C and Benthic-Atmosphere (B_atm_) ages in order to understand the ventilation dynamics of the equatorial Indian Ocean thermocline during the Last Glacial Maximum (LGM). Our results demonstrate a radiocarbon depleted thermocline as low as -250 to -345‰ (∆∆^14^C), corresponding to ~ 500–2100 years (B_atm_) old waters at the LGM compared to ~ 380 years today. More broadly, we show that thermocline ventilation ages are one order of magnitude more variable than previously thought. Such a radiocarbon depleted thermocline can at least partly be explained by variable abyssal upwelling of deep-water masses with elevated respired carbon concentrations. Our results therefore have implications for radiocarbon-only based age models and imply that upper thermocline waters as shallow as 400 m depth can also contribute to some of the glacial carbon drawdown.

## Introduction

Ocean circulation and ocean ventilation are crucial drivers of Earth’s climate system. Ocean ventilation is the process by which surface waters, recently in contact with the atmosphere, are injected into the ocean interior and transported away from the source (aging of water masses). Solubility, biological, and alkalinity pumps are the main mechanisms that foster the storage of roughly 50 times more carbon in the deep-sea compared to the atmosphere^1,2^, making the deep-sea the largest active Earth surface carbon reservoir. Accordingly, Quaternary glacial and interglacial variations in atmospheric CO_2_ concentrations have been attributed to changes in the sink and source properties of the Earth surface carbon cycle, particularly to the marine carbon cycle^3–5^. Although there appears to be some heterogeneity in the different sectors of the Southern Ocean^6–8^, there is growing evidence for the presence of an isolated carbon reservoir during the last glacial period that may have accumulated re-mineralized (respired) organic carbon, ^8–14^. In addition, it has been suggested that the deep ocean possibly absorbed 730–980 Pg of dissolved inorganic carbon (DIC) during the Last Glacial Maximum (LGM, 23–19 ka) of which one third may be accounted for by a transfer from thermocline and intermediate waters^15^. This demonstrates that the deep-sea carbon inventory is heterogeneous. It is well admitted that the deep-sea carbon pool did age significantly during the LGM, but there is a profound uncertainty regarding the exchange between surface and deep ocean, i.e. the role of intermediate and thermocline waters.

Reconstructions of thermocline and intermediate water ventilation are still sparse and often exhibit conflicting results^14–22^, leaving their importance in particular enigmatic. Inconsistencies in glacial and deglacial ventilation records may at least partly be the result of using foraminiferal radiocarbon dates relative to the atmosphere. Cold-water corals (CWC) have been shown to serve as a robust archive for several geochemical proxies^23–30^ in particular for the thermocline and deeper waters. Their aragonite skeletons contain relatively high concentrations of uranium, allowing accurate age determination through ^230^Th/U dating^26^. Combined ^230^Th/U and ^14^C measurements enable us to determine past ocean ^14^C/^12^C ratios and thus are a proxy for accurate and precise ∆^14^C and corresponding ventilation ages^29,30^. Moreover, CWC aggradations often occur near the boundaries of thermocline and intermediate waters, which are sensitive to large-scale oceanographic perturbations^31^.

**Figure 1:**
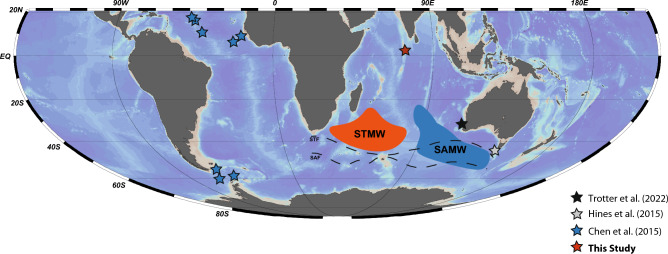
World map showing the different cold-water coral locations that have been analysed by paired ^230^Th/U and ^14^C measurements and are presented here: the equatorial Atlantic^10^ (750–1492 m water depth), off Tasmania^35^ (1430–1950 m), off W-Australia^23^, (675–1788 m), Drake Passage^10^ (750–2100 m) as well as off the Maldives (this study, 450 m). Also shown are the source regions of the Subtropical Mode Water (red) and Subantarctic Mode Water (blue) according to ref^63^ as well as the Suptropical Front (STF) and Subantartic Front (SAF).The map was generated with the webODV Explore^64^.

**Figure 2:**
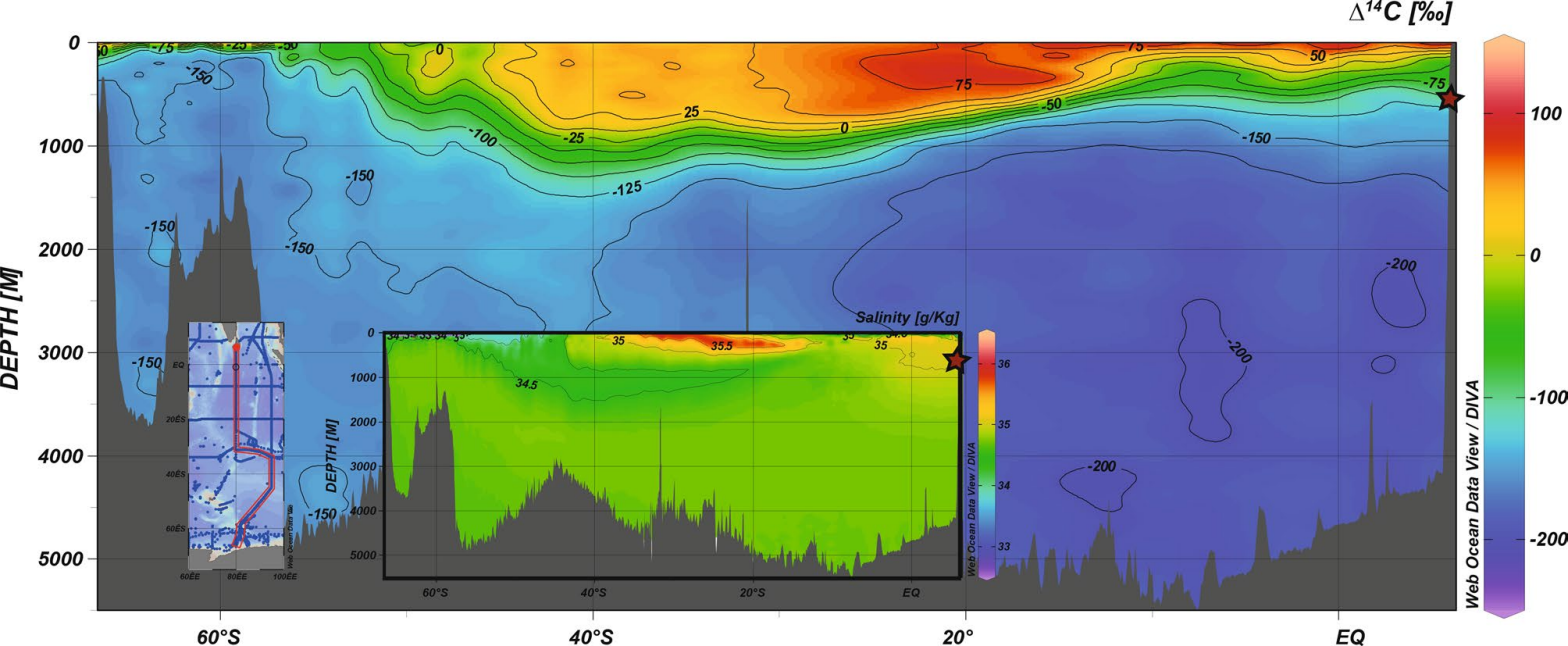
Modern distribution of seawater ∆^14^C in the Indian Ocean. Position of the chosen section is shown in the small map (left). Also shown is on the same section the salinity distribution. Data was taken from the global dataset GLODAP v2. ^65,66^ and plotted with webODV Explore^64^. The red stars indicate the sampling site of this study.

Here we present the first ventilation ages derived from paired ^230^Th/U and ^14^C measurement on CWCs retrieved from thermocline waters (here: the permanent thermocline is the transitional zone where surface and deep waters mix) at 450 m water depth off the Maldives in the equatorial Indian Ocean^32^ (Fig. 1). These measurements allow for the determination of ∆^14^C and Benthic-Atmospheric (B_atm_) ^14^C ventilation ages for each sample (based on IntCal20^33^). Finally, we compare our results with glacial radiocarbon simulations applying an ocean general circulation model including ∆^14^C^32^. The Indian Ocean is only ventilated from the south and thus a cul de sac, making it an important compartment for thermohaline circulation and especially for reconstructing past ∆^14^C of thermocline waters originating in the Southern Oceans (Fig. 2). Our new coral data provides answers to questions regarding how variable equatorial Indian Ocean thermocline ventilations ages have been and whether they have contributed to the drawdown of carbon during the LGM.

## Coupled ^230^Th/U and ^14^C of cold-water corals from the Maldives

**Figure 3:**
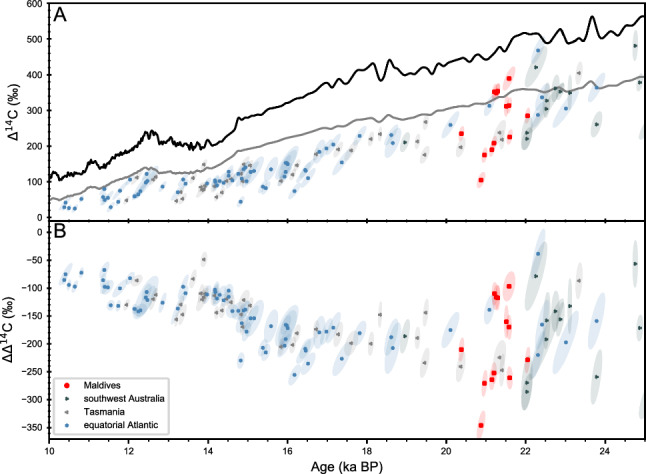
(**A**) ∆^14^C records from the equatorial Atlantic^10^, Tasmania^35^, and southwest Australia^23^ and from the Maldives (this study, from the shallowest water depth of 450 m). For comparison, the ∆^14^C record are plotted against the calibration curves IntCal20^33^ (black) and Marine20^34^ (grey), (**B**) Calculated ∆∆^14^C (∆^14^C against atmospheric ∆^14^C at the respective interval) plotted together with the above-mentioned records. Note we only plot ∆^14^C and ∆∆^14^C reconstructions that are based on paired ^230^Th/U and ^14^C analyses. Note we have taken out one data point at ~ 17.5 by ref^23^ that exhibits an extremely large error in ∆^14^C and corresponding ∆∆^14^C.

In accordance to previous results^32^, our ^230^Th/U age determinations demonstrate that the analysed CWCs fall into a very narrow time interval close to the LGM with a minimum age of 20.413 ± 0.059 ka and maximum age of 22.096 ± 0.055 ka (Table 1s).The corresponding radiocarbon ages (calibrated against IntCal20^33^) are systematically older and reveal ages from 22.071 ka to 23.503 ka (Fig. 2, Table 2s). Our dataset reveals two prominent features. Firstly, Indian Ocean thermocline waters off the Maldives in 450 m water depth appear to be extremely variable with a range in the calculated ∆^14^C between + 109‰ and + 392‰ within a time span of less than 1.5 ka. Secondly, they are depleted compared to the IntCal20^33^ atmospheric ^14^C curve, and most of the (surface) Marine20^34^ curve at their corresponding calendar ages (Fig. 3). This ^14^C depletion of thermocline water is shown in ∆∆^14^C (i.e. the ∆^14^C difference between atmosphere and corals) values as low as − 250‰ to − 345‰ , corresponding to B_atm_ ages of up to 2100 years. The observed variability in B_atm_ ages cannot be related to species, but tend to cluster with higher B_atm_ ages at the slightly deeper site (Malé Vaadhoo channel), although both are only < 100 km apart from each other and are both located on the eastern side of the Maldives.

**Figure 4:**
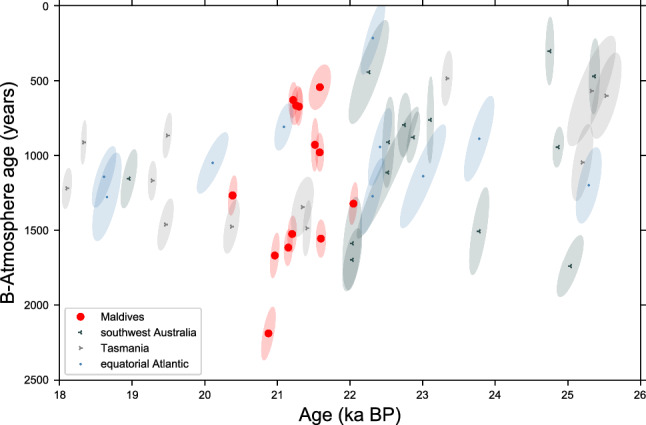
(**A**) Benthic–Atmosphere ages (ventilation ages) records from ref^10,23,35^ and this study at shallowest water depth of 450 m based on the calculated ∆∆^14^ values, calibrated against IntCal20^33^. Note we only plot B_atm_ age reconstructions that are based on paired ^230^Th/U and ^14^C analyses.

Moreover, our ∆∆^14^C and B_atm_ values for the Indian Ocean thermocline at the LGM are similar to those observed off Tasmania in far deeper water depths of 1430–1950 m, off SW-Australia in water depth as deep as 1788 m and from the Drake Passage (750–2100 m water depth^10,23,35,36^, Figs. 1, 3 and 4). Note that presently these water masses have moderately lower (− 25‰ to − 50‰ ) seawater ∆^14^C values as compared to the pre-bomb thermocline waters of the Indian Ocean^37,38^. Furthermore, the thermocline ∆∆^14^C values and B_atm_ ages off the Maldives during the LGM are more depleted in radiocarbon as intermediate to deep-water masses in the equatorial Atlantic^10^, and are more depleted compared to modern and Holocene reservoir ages in source region of the SAMW ^39,40^ (Figs. 3 and 4).

## Comparison with model results

**Figure 5:**
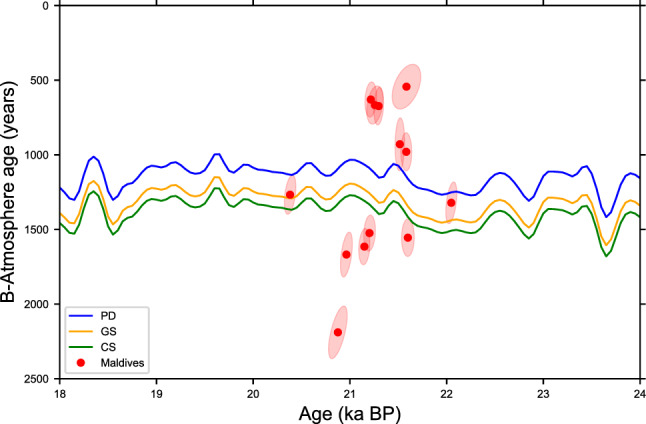
Comparison of radiocarbon model simulations and the reconstructed B_atm_ ages. Model simulations are depicted in blue for PD (present control), yellow for GS (glacial ocean) and green for CS (glacial stadial). For details, please see text. Reconstructed ∆∆^14^C values are shown the supplementary material.

For further analysis, we simulated the temporal evolution of radiocarbon in the equatorial thermocline. Our coral based ∆∆^14^C (supplementary material) values and B_atm_ ages broadly agree with the radiocarbon simulations but some discrepancies are visible (Fig. 5 and 2s). In particular, the temporal variability of simulated B_atm_ ages is considerably smaller than reconstructed. Simulated B_atm_ ages vary roughly from 1000 to 1500 years in the interval covered by the corals, whereas the corals exhibit a B_atm_ range from 500 to 2300 years. Correspondingly, within an interval of less than 1.5 ka, the coral based B_atm_ ages are outside the uncertainty bounds spanned by the various model scenarios (Fig. 5, ∆∆^14^C in the supplementary material).

This indicates that the simulations underestimate the past radiocarbon variability of the Indian Ocean thermocline. While previous ^14^C simulations for the LGM were roughly consistent with benthic ^14^C values reconstructed on other locations^41^, our new data from the Indian Ocean highlights that the ^14^C history of glacial thermocline waters is complex. Thermocline waters are at the transient zone between the surface mixed layer and the deep-ocean. Especially in the glacial ocean, where deeper waters stored additional radiocarbon depleted carbon^8,9,11^, the equatorial thermocline of the Indian Ocean tends to reflects both atmospheric ∆^14^C and deep ocean ∆^14^C. However, even if radiocarbon depleted but carbon rich deep-water reservoirs are a pervasive feature of the glacial ocean, high ventilation ages in near surface waters are a difficult phenomenon to explain. In the following, we consider three hypotheses that could explain the variability and depletion of ^14^C reconstructed for the glacial thermocline of the Indian Ocean: (1) in-situ aging, (2) advection of ^14^C-depleted mid-depth water masses, as well as (3) local upward mixing of ^14^C-depleted carbon.

## In situ aging

Lowest ventilation ages recorded in our dataset plot near or in-between the Intcal20^33^ and Marine20^34^ ∆^14^C curves as expected for thermocline water masses that have been in contact with the atmosphere. However, the observed variability in ventilations ages suggest a strong but variable aging of thermocline waters. Can the observed radiocarbon decline be explained by an in-situ aging from an isolated thermocline water? We discount this hypothesis for the following reasons, (a) our sites here are not horizontally isolated from other ocean basins, (b) in-situ aging is at odds with the amplitude and rapidity of our reconstructed ∆∆^14^C variations (about 300‰ within 1500 years).

## Advection of intermediate and mode waters

The decadal to centennial scale variability seen in our ^14^C record could be explained by the advection of southern sourced mode waters^16,17^. Here, the principal mechanism is the upwelling of carbon and nutrient-rich water in the Southern Ocean, which is subsequently transported to the equatorial thermocline by the Antarctic Intermediate Water (AAIW) and the Subantarctic Mode Water (SAMW)^16,17^. In the Equatorial Pacific, the advection of such Southern Ocean radiocarbon depleted waters was synchronous with deep-water ventilation changes^22^. However, even though this mechanism has been proposed for periods of abrupt climatic perturbations such as the Younger Dryas and Heinrich Stadials I and II, reconstructed ventilation ages of the intermediate northern Indian Ocean do not exhibit any larger excursions during the LGM^17^. Further evidence comes from a neodymium isotope based reconstructions showing, that advances of AAIW in the equatorial Indian Ocean are restricted to the deglaciation and did not occur during the LGM^42^.

It has been suggested that increased glacial reservoir ages could be related to decreased air-sea equilibration during the LGM^43^. However, the amplitude of our reconstructed ventilation changes rather supports the hypothesis of altered glacial deep-sea overturning and increased CO_2_ storage, as recently suggested by a comprehensive compilation of glacial deep-sea ^14^C records^11^.

Nevertheless, with the present dataset we cannot rule out that radiocarbon depleted mid-depth waters, either SAMW or AAIW, may have partly contributed to the observed variability in the thermocline ventilations ages.

## Abyssal upward mixing of ^14^C depleted carbon

**Figure 6:**
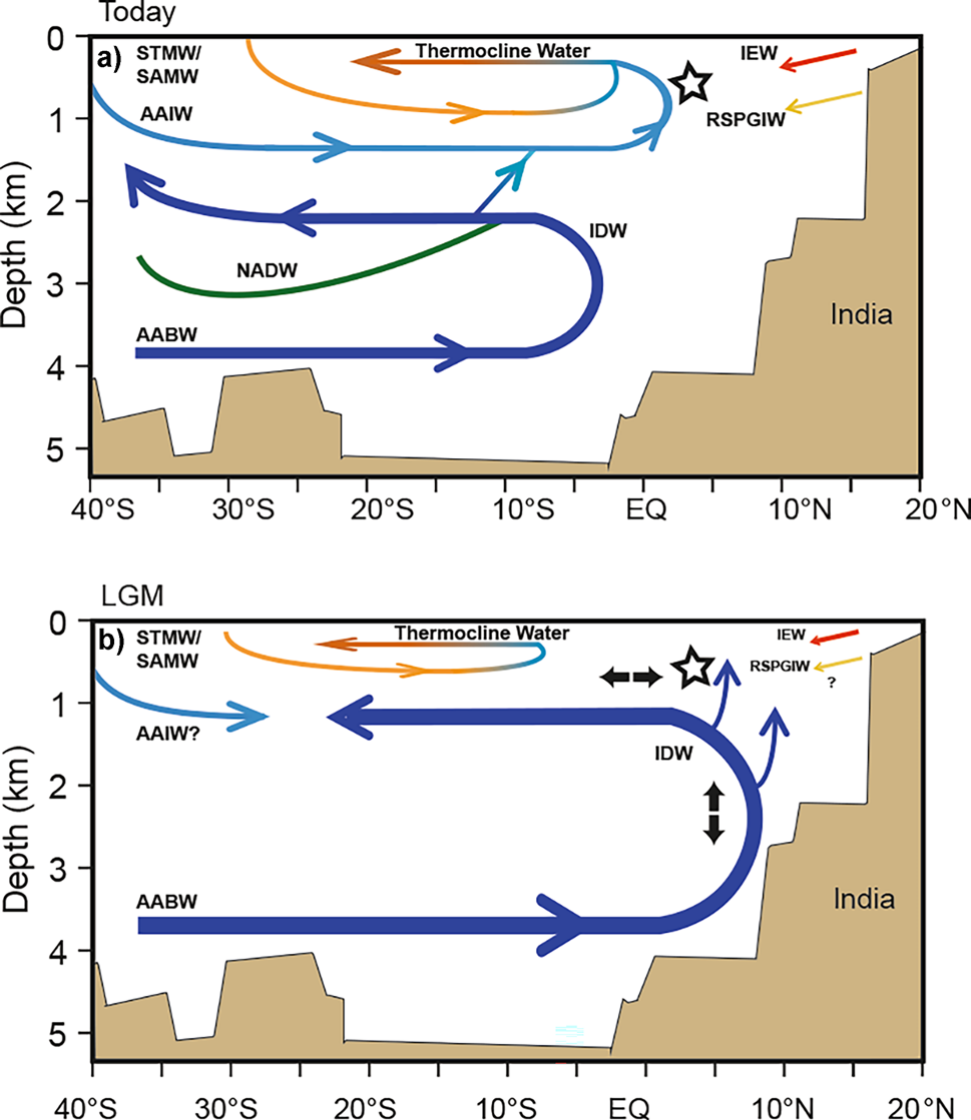
Simplified ocean circulation sketch of the modern (a) and LGM (b) Indian Ocean. Modern circulation pattern is based on ref^44,46^ (top panel). The modern circulation in the Indian Ocean is characterised by inflowing Antarctic Bottom Water (AABW) at depth, and inflowing shallower North Atlantic Deep-Water (NADW, green). Towards the Indian continent, AABW diffuses upward and flows back southward as the Indian Deep Water (IDW) above the NADW. Above the IDW, southern sourced mid-depth waters such as Antarctic Intermediate Water and Sub Antarctic Mode Water penetrates into the lower latitudes. In thermocline waters (TW), surface and deep waters mix. During the LGM, our ventilation ages suggest that the AABW and the IDW episodically diffused upward and thereby potentially reduced advances of southern sources mid-depth waters and expanded into TW, although with high temporal variability. Also shown are near surface waters originating in the Northern Indian Ocean such as the Indian Equatorial Water (IEW) and the Red Sea–Persian Gulf Intermediate Water (RSPGIW)^67^.

Our reconstructed variable and increased ventilation ages of thermocline waters in the equatorial Indian Ocean during the LGM can be attributed to upward mixing of deep waters. As the Indian Ocean is solely ventilated from the south^44^, modern Indian Deep Water (IDW) is formed from abyssal waters such as Antarctic Bottom Water via diapycnal mixing in the interior^37–45^^.^, thereby increasing the volume of southern sourced water masses at shallower water depths (Figs. 2 and 6). Thus, abyssal upwelling controls the distribution pattern of DIC and ^14^C concentrations, revealing a gradual aging that ends up in the upper deep-water of the northern Indian Ocean^45–49^. Consequently, modern IDW is considered as a key supplier of carbon for the Southern Ocean upwelling^37^. During the last glacial period and in particular during the LGM, southern sourced waters expanded into deep and abyssal depth of the Indian Ocean, displaced the ambient Atlantic source water mass and thereby significantly increased the carbon storage capacity of the deep^39,50–52^. A replacement by a southern sourced deep-water mass could therefore be accompanied by poor ventilation and in turn by a lack of oxygen replenishment. As a water mass remains isolated from the atmosphere, ^14^C decays while oxygen is consumed due to oxidation of organic matter. Thus, we would expect water mass aging to be accompanied by decreasing oxygen concentrations. Indeed, there is evidence for anoxic bottom waters during the LGM in the deep Indian Ocean^49,52^. Accordingly, poorly ventilated deep-water masses and anoxic conditions point towards an extremely radiocarbon depleted deep-water in the abyssal and deep Indian Ocean, which may have extended into the thermocline leading to temporally very variable ventilation ages.

During the LGM, radiocarbon depleted but carbon rich waters have been identified in the Southern Ocean such as the Drake Passage, in the Indian Sector of the Southern Ocean as well as off Tasmania, but with B_atm_ ages lower than < 3000 years^10,35,36^. Mid-depth waters tend to shoal on the pathway into the tropics^44^. Thus, a ^14^C Southern Ocean signal would be diluted with ^14^C enriched low-latitude surface waters during the pathway into the Indian Ocean, making it difficult to generate B_atm_ ages of up to ~ 2100 years in the equatorial thermocline. This would in turn imply that a substantially older deep-water mass is required to explain the observed ^14^C depletion in thermocline waters. Very high ventilation ages near the LGM (> 4000 years) have been identified in the (SW) Pacific Ocean^8,14,39^, but also in the northern deep and abyssal Indian Ocean by using fossil foraminiferal ∆^14^C ages^51,53^. These extremely old deep- and abyssal water masses may thus be the most likely potential radiocarbon depleted source to cause, by upward mixing with the overlying water mass, the accumulation of ^14^C-depleted DIC in the equatorial thermocline of the Indian Ocean (Fig. 6).

Moreover, these radiocarbon depleted thermocline waters at the LGM may have also contributed to the deglacial release (Younger Dryas and Heinrich Stadial 1) of ^14^C depleted intermediate water masses in the Arabian Sea^17^, implying strong local differences of carbonate system characteristics.

Taken together, our new equatorial thermocline Indian Ocean ^14^C data points towards extensive, but variable mixing of the Indian Ocean equatorial thermocline with extremely ^14^C-depleted abyssal waters. Our study therefore shows that the deep Indian Ocean carbon reservoir, although temporally restricted, expanded to thermocline waters and thus contributed to the drawdown of atmospheric CO_2_ at the end of the last glacial period. The dynamic nature of this oceanographic phenomenon suggest that this extended carbon pool is regionally variable. Accordingly, future studies should intensively try to identify regional differences and depth constraints of carbon pool extension especially in the Indian Ocean.

## Online methods

### Cold-water coral samples

This study analysed scleractinian cold-water corals retrieved during research cruise SO236 to the Maldives Archpielago. In particular, coral samples were collected by a video-guided grab and a box corer in the Vaadhoo Channel (SO236-007, 04°09.07ʹN, 73°29.28ʹE, 443 m water depth) and the Kardiva Channel (SO236-017-TVG, 04°51.26N, 73°28.05ʹE, 453–457 m water depth. Initial radiocarbon datings^32^ revealed calibrated ages near the LGM between 22.54 and 21.4 ka. Thus, this sample set provides the unique opportunity to study ventilation ages at thermocline depth of the equatorial Indian Ocean during the LGM. Well-preserved coral skeletons (*Desmophyllum pertusum*, *Enallopsammia rostrata* and *Madrepora oculata*) were cleaned mechanically in order to remove potential containments (*e.g.*, ferro-manganese coatings, borings, epibionts). Samples have been screened for their mineralogy with a PANalytical X’Pert PRO diffractometer, equipped with a copper X-ray tube revealing that all samples remained in their initial aragonitic mineralogy.

### ^230^Th/U ages determinations

Samples were chemically cleaned in a weak acid leach^28^. The ^230^Th/U measurements were carried out at the Institute of Environmental Physics at Heidelberg University (IUP, Germany) on a multi-collector inductively coupled plasma mass spectrometer (ThermoFisher, Neptune Plus)^28^. The reference material HU-1 was measured for the reproducibility assessment of the mass-spectrometry measurements^54^. Note, we assume HU-1 to be in secular equilibrium, which contrasts with observations by ref^54^ and causes a 1.5‰ difference in the absolute value of δ^234^U. For age determination this difference has no consequence, as we use the half-lives of ref^54^ for age determination, hence we presume a different isotopic composition for our batch of HU-1 if compared to the data published by ref^54^. In total, 13 samples were analysed revealing all only minor residual contaminations (^232^Th < 4 ppb). Nevertheless, an initial ^230^Th correction was applied prior to age calculations using a ^230^Th/^232^Th activity ratio for the upper thermocline waters of 8 ± 4^18^. Age determinations and uncertainty assessment were carried out using iterative solution of the decay equations and error propagation using Monte Carlo simulations^26^. The initial ^234^U/^238^U activity ratios of all measured corals are, when transferred into δ^234^U notation (i.e., ‰ deviation from secular equilibrium), within uncertainty in a narrow band of ± 10‰ compared to the value of modern seawater (145.0 ± 1.5‰^55^), suggesting a closed system behaviour for the exchange of U between the skeletons and seawater.

### Radiocarbon measurements

The extraction of CO_2_ from the CWC samples was carried out at the IUP, Heidelberg University, Germany, following the method described in^56^. The final iron–graphite compound was measured on an accelerator mass spectrometer (AMS, MICADAS) at the Curt-Engelhorn-Center Archaeometry (CEZA), Mannheim, Germany^57,58^. Calculation of ∆^14^C, ∆∆^14^C and Benthic-Atmosphere (B_atm_) ages ^10,29,30^ is based on IntCal20^33^.

### Modelling

The radiocarbon measurements were compared with ∆^14^C values simulated using an enhanced version of the Hamburg Large Scale Geostrophic ocean general circulation model^59^; for the enhancements and implementation of ∆^14^C see refs.^60,61^ and further references therein. The model has an effective horizontal resolution of 3.5° and 22 layers in the vertical. It considers recent (PD), cold stadial (CS) and glacial (GS) climatic background conditions which result in upper and lower ocean ventilation intensities. The simulations were carried out with transient values of atmospheric ∆^14^C^34^ and *p*CO_2_^62^ evaluated nearest to the coral sites.

## Supplementary Information

Supplementary Figures.

Supplementary Table 1.

Supplementary Table 2.

## Data Availability

Data associated to this article can be found in the supplementary online material.

## References

[1] Toggweiler JR (1999). Variation of atmospheric CO2 by ventilation of the ocean's deepest water. Paleoceanography.

[2] Zeebe RE (2012). History of seawater carbonate chemistry, atmospheric CO_2_, and ocean acidification. Annu. Rev. Earth Planet. Sci..

[3] Sigman D, Boyle E (2000). Glacial/interglacial variations in atmospheric carbon dioxide. Nature.

[4] Robinson LF (2005). Radiocarbon variability in the western North Atlantic during the last deglaciation. Science.

[5] Skinner LC (2017). Radiocarbon constraints on the glacial ocean circulation and its impact on atmospheric CO_2_. Nat. Commun..

[6] Gottschalk J, Michel E, Thöle LM (2020). Glacial heterogeneity in Southern Ocean carbon storage abated by fast South Indian deglacial carbon release. Nat Commun..

[7] Skinner LC, Freeman E, Hodell D, Waelbroeck C, Riveiros NV, Scrivner AE (2021). Atlantic Ocean ventilation changes across the last deglaciation and their carbon cycle implications. Paleoceanogr. Paleoclimatol..

[8] Sikes EL, Samson CR, Guilderson TP, Howard WR (2000). Old radiocarbon ages in the southwest Pacific Ocean during the last glacial period and deglaciation. Nature.

[9] Skinner LC, Fallon S, Waelbroeck C, Michel E, Barker S (2010). Ventilation of the deep Southern Ocean and deglacial CO_2_ rise. Science.

[10] Chen T, Robinson LF, Burke A, Southon J, Spooner P, Morris PJ, Ng HC (2015). Synchronous centennial abrupt events in the ocean and atmosphere during the last deglaciation. Science.

[11] Rafter PA (2022). Global reorganization of deep-sea circulation and carbon storage after the last ice age. Sci. Adv..

[12] Rae JWB, Burke A, Robinson LF, Adkins JF, Chen T, Cole C, Greenop R, Li T, Littley EFM, Nita DC, Stewart JA, Taylor BJ (2018). CO_2_ storage and release in the deep Southern Ocean on millennial to centennial timescales. Nature.

[13] Gottschalk J, Skinner L, Lippold J (2016). Biological and physical controls in the Southern Ocean on past millennial-scale atmospheric CO_2_ changes. Nat. Commun..

[14] Ronge TA, Prange M, Mollenhauer G, Ellinghausen M, Kuhn G, Tiedemann R (2020). Radiocarbon evidence for the contribution of the Southern Indian ocean to the evolution of atmospheric CO_2_ over the last 32,000 years. Paleoceanogr. Paleoclimatol..

[15] Sarnthein M, Schneider B, Grootes PM (2013). Peak glacial ^14^C ventilation ages suggest major draw-down of carbon into the abyssal ocean. Clim. Past.

[16] Marchitto TM, Lehman SJ, Ortiz JD, Flückiger J, van Geen A (2007). Marine radiocarbon evidence for the mechanism of deglacial atmospheric CO_2_ rise. Science.

[17] Bryan SP, Marchitto TM, Lehman SJ (2010). The release of C-14-depleted carbon from the deep ocean during the last deglaciation: Evidence from the Arabian Sea. Earth Planet. Sci. Lett..

[18] Mangini A (2010). Deep-sea corals off Brazil verify a poorly ventilated Southern Pacific Ocean during H2, H1 and the younger Dryas. Earth Planet. Sci. Lett..

[19] De Pol-Holz R, Keigwin L, Southon J, Hebbeln D, Mohtadi M (2010). No signature of abyssal carbon in intermediate waters off Chile during deglaciation. Nat. Geosci..

[20] Duplessy JC (1989). AMS C-14 study of transient events and of the ventilation rate of the Pacific intermediate water during the last deglaciation. Radiocarbon.

[21] Curry WB, Oppo DW (2005). Glacial water mass geometry and the distribution of delta C-13 of Sigma CO_2_ in the western Atlantic Ocean. Paleoceanography.

[22] Umling N, Thunell R (2017). Synchronous deglacial thermocline and deep-water ventilation in the eastern equatorial Pacific. Nat. Commun..

[23] Trotter J (2022). Deep-water coral records of glacial and recent ocean-atmosphere dynamics from the Perth Canyon in the southeast Indian Ocean. Quat. Sci. Adv..

[24] Montagna P (2014). Li/Mg systematics in scleractinian corals: Calibration of the thermometer. Geoch. Cosmochim. Acta.

[25] Robinson LF (2014). The geochemistry of deep-sea coral skeletons: a review of vital effects and applications for palaeoceanography. Deep Sea Res. Part II Top Stud. Oceanogr..

[26] Raddatz J, Rüggeberg A (2021). Constraining past environmental changes of cold-water coral mounds with geochemical proxies in corals and foraminifera. Depos. Rec..

[27] Davies A (2022). Dual clumped isotope thermometry of coral carbonate. Geoch. Cosmochim. Acta.

[28] Wefing AM, Arps J, Blaser P, Wienberg C, Hebbeln D, Frank N (2017). High precision U-series dating of scleractinian cold-water corals using an automated chromatographic U and Th extraction. Chem. Geol..

[29] Adkins JF, Cheng H, Boyle EA, Druffel ERM, Edwards RL (1998). Deep-sea coral evidence for rapid change in ventilation of the deep North Atlantic 15,400 years ago. Science.

[30] Mangini A (1998). Coral provides way to age deep water. Nature.

[31] Rüggeberg A, Flögel S, Dullo W-C, Raddatz J, Liebetrau V (2016). Paleoseawater density reconstruction and its implication for cold-water coral carbonate mounds in the northeast Atlantic through time. Paleoceanography.

[32] Reolid J, Reolid M, Betzler C (2017). Upper Pleistocene cold-water corals from the inner sea of the Maldives: Taphonomy and environment. Facies.

[33] Reimer PJ (2020). The IntCal20 Northern Hemisphere radiocarbon age calibration curve (0–55 cal kBP). Radiocarbon.

[34] Heaton TJ (2020). Marine20—the marine radiocarbon age calibration curve (0–55,000 cal BP). Radiocarbon.

[35] Hines SKV, Southon JR, Adkins JF (2015). A high-resolution record of Southern Ocean intermediate water radiocarbon over the past 30000 years. Earth Planet. Sci. Lett..

[36] Burke A, Robinson LF (2012). The Southern Ocean’s role in carbon exchange curing the last deglaciation. Science.

[37] Key RM (2004). A global ocean carbon climatology: Results from global data analysis project (GLODAP). Glob. Biogeochem. Cyc..

[38] Southon J, Kashgarian M, Fontugne M, Metivier B, Yim W (2002). Marine reservoir corrections for the Indian Ocean and southeast Asia. Radiocarbon.

[39] Sikes EL, Cook MS, Guilderson TP (2016). Reduced deep ocean ventilation in the Southern Pacific Ocean during the last glaciation persisted into the deglaciation. Earth Planet. Sci. Lett..

[40] Komugabe AF, Fallon SJ, Thresher RE, Eggins SM (2014). Modern Tasman Sea surface reservoir ages from deep-sea black corals. Deep-Sea Res..

[41] Butzin M, Prange M, Lohmann G (2005). Radiocarbon simulations for the glacial ocean: The effects of wind stress, Southern Ocean sea ice and Heinrich events. Earth Planet. Sci. Lett..

[42] Yu Z (2018). Antarctic intermediate water penetration into the northern Indian Ocean during the last deglaciation. Earth Planet Sci. Lett..

[43] Galbraith ED, Skinner LC (2020). The biological pump during the last glacial maximum. Annu. Rev. Mar. Sci..

[44] Talley LD (2013). Closure of the global overturning circulation through the Indian, Pacific, and Southern Oceans: Schematics and transports. Oceanography.

[45] Schmitz WJ (1995). On the interbasin-scale thermohaline circulation. Rev. Geophys..

[46] Talley LD, Pickard GE, Emery WJ, Swift JH (2011). Descriptive Physical Oceanography: An Introduction.

[47] Matsumoto K (2007). Radiocarbon-based circulation age of the world oceans. J. Geophys. Res..

[48] Srinivasan A, Rooth C, Top Z, Olson DB (2000). Abysal upwelling in the Indian Ocean: Radiocarbon diagnostics. J. Mar. Res..

[49] Sarkar A, Bhattacharya SK, Sarin MM (1993). Geochemical evidence for anoxic deep water in the Arabian Sea during the last deglaciation. Geochem. Cosmochim. Acta.

[50] Piotrowski AM, Banakar VK, Scrivner AE, Elderfield H, Galy A, Dennis A (2009). Indian ocean circulation and productivity during the last glacial cycle. Earth Planet Sci. Lett..

[51] Bharti N, Bhushan R, Skinner L, Muruganantham M, Jena PS, Dabhi A, Shivam A (2022). Evidence of poorly ventilated deep Central Indian Ocean during the last glaciation. Earth Planet Sci. Lett..

[52] Chandana KR, Bhushan R, Jull AJT (2017). Evidence of Poor bottom water ventilation during LGM in the equatorial Indian Ocean. Front. Earth Sci..

[53] Nisha K, Naik SS, Kumar P, Banerjee B, Murty PDR (2023). Radiocarbon evidence for reduced deep water ventilation of the northern Indian Ocean during the last glacial maxima and early deglaciation Earth Planet. Sci. Lett..

[54] Cheng H (2013). Improvements in 230Th dating, 230^Th^ and 234U half-life values, and U-Th isotopic measurements by multi-collector inductively coupled plasma mass spectrometry. Earth Planet Sci. Lett..

[55] Chutcharavan PM, Dutton A, Ellwood MJ (2018). Seawater 234U/238U recorded by modern and fossil corals. Geoch. Cosm. Acta.

[56] Therre S, Proß L, Friedrich R, Trüssel M, Frank N (2021). Heidelberg radiocarbon lab–establishing a new carbon dioxide extraction line for carbonate samples. Radiocarbon.

[57] Synal H-A, Stocker M, Suter M (2007). MICADAS: A new compact radiocarbon AMS system. Nucl. Instrum. Meth. Phys. Res. B Nucl. Instrum. Meth. B.

[58] Kromer B, Lindauer S, Synal H-A, Wacker L (2013). MAMS–a new AMS facility at the Curt-Engelhorn-Centre for Archaeometry, Mannheim, Germany. Nucl. Instrum. Methods Phys. Res. B Nucl. Instrum. Meth. B.

[59] Maier-Reimer E, Mikolajewicz U, Hasselmann K (1993). Mean circulation of the Hamburg LSG OGCM and its sensitivity to the thermohaline surface forcing. J. Phys. Oceanogr..

[60] Butzin M, Köhler P, Lohmann G (2017). Marine radiocarbon reservoir age simulations for the past 50,000 years. Geophys. Res. Lett..

[61] Butzin M, Heaton TJ, Köhler P, Lohmann G (2020). A short note on marine reservoir age simulations used in IntCal20. Radiocarbon.

[62] Köhler P, Nehrbass-Ahles C, Schmitt J, Stocker TF, Fischer H (2017). A 156 kyr smoothed history of the atmospheric greenhouse gases CO_2_, CH_4_, and N_2_O and their radiative forcing. Earth Syst. Sci. Data.

[63] Koch-Larrouy A, Morrow R, Penduff T, Juza M (2020). Origin and mechanism of Subantarctic Mode Water formation and transformation in the Southern Indian Ocean. Ocean Dyn..

[64] Schlitzer, R., Mieruch-Schnülle, S. webODV Explore, https://explore.webodv.awi.de, (2021).

[65] Lauvset SK, Lange N, Tanhua T, Bittig HC, Olsen A, Kozyr A, Álvarez M, Becker S, Brown PJ, Carter BR, da Cunha CL, Feely RA, van Heuven S, Hoppema M, Ishii M, Jeansson E, Jutterström S, Jones SD, Karlsen MK, Lo Monaco C, Michaelis P, Murata A, Pérez FF, Pfeil B, Schirnick C, Steinfeldt R, Suzuki T, Tilbrook B, Velo A, Wanninkhof R, Woosley RJ, Key RM (2021). An updated version of the global interior ocean biogeochemical data product, GLODAPv2.2021. Earth Syst. Sci. Data.

[66] Lauvset SK, Lange N, Tanhua T, Bittig HC, Olsen A, Kozyr A, Alin S, Álvarez M, Azetsu-Scott K, Barbero L, Becker S, Brown PJ, Carter BR, da Cunha LC, Feely RA, Hoppema M, Humphreys MP, Ishii M, Jeansson E, Jiang L-Q, Jones SD, Lo Monaco C, Murata A, Müller JD, Pérez FF, Pfeil B, Schirnick C, Steinfeldt R, Suzuki T, Tilbrook B, Ulfsbo A, Velo A, Woosley RJ, Key RM (2022). GLODAPv2.2022: The latest version of the global interior ocean biogeochemical data product. Earth Syst. Sci. Data.

[67] Harms NC, Lahajnar N, Gaye B, Rixen T, Dähnke K, Ankele M, Schwarz-Schampera U, Emeis K-C (2019). Nutrient distribution and nitrogen and oxygen isotopic composition of nitrate in water masses of the subtropical southern Indian Ocean. Biogeosciences.

