# Supplementary material for: Variable ventilation ages in the equatorial Indian Ocean thermocline during the LGM
Source: Sci Rep. 2023 Jul 13;13:11355. doi: 10.1038/s41598-023-38388-z (PMC10345116; doi:10.1038/s41598-023-38388-z)
Supplement: Supplementary file 1 — Supplementary Figures. [file 41598_2023_38388_MOESM1_ESM.docx]

**Supplementary Material**


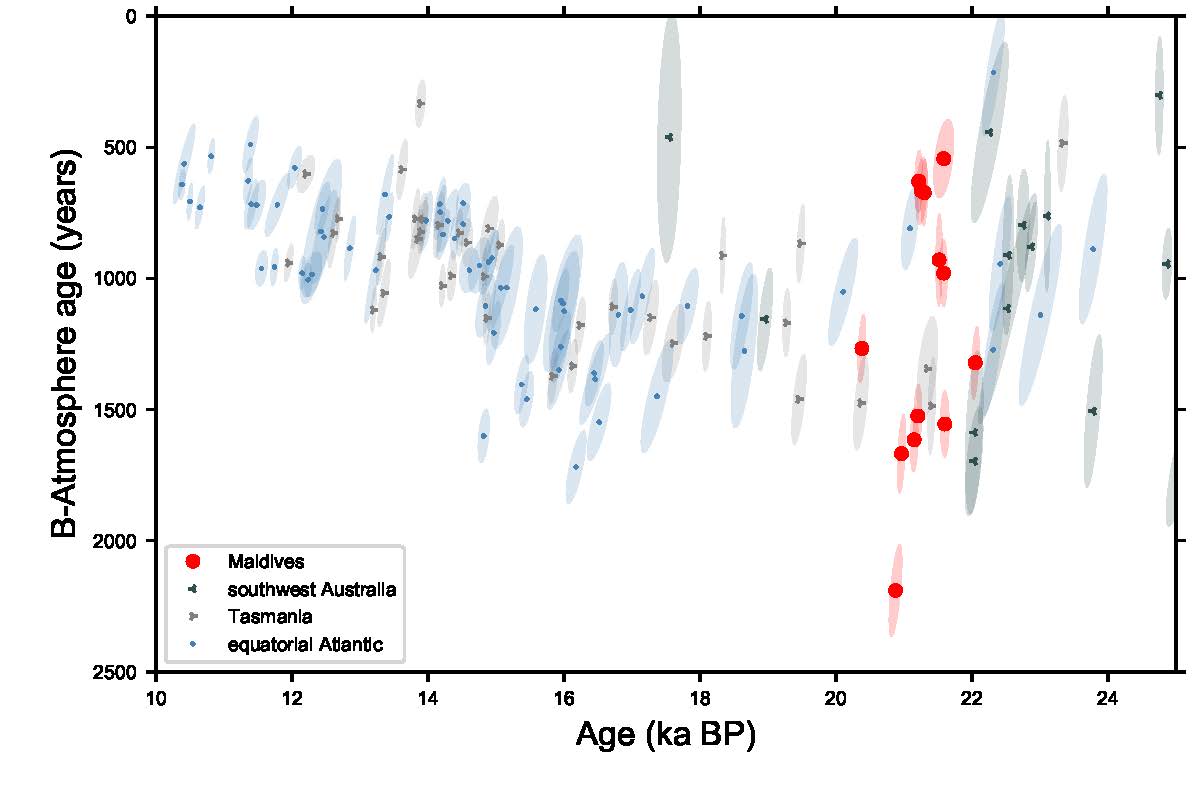


**Figure 1s:** Same as figure 4, but with an extended age scale Reconstructed B_atm_ ages (ventilation ages) are the difference between the absolute ^230^Th/U age and the ^14^C age of each individual coral based on IntCal20^33^) Also calculated are the B_atm_ ages of the above mentioned coral based studies. Data of ref ^10,23,35^ are plotted for comparison. Note we only plot B_atm_ age reconstructions that are based on paired ^230^Th/U and ^14^C analyses. Note we have taken out one data point at 17.5 by ref 23, that exhibits an extremely large error.


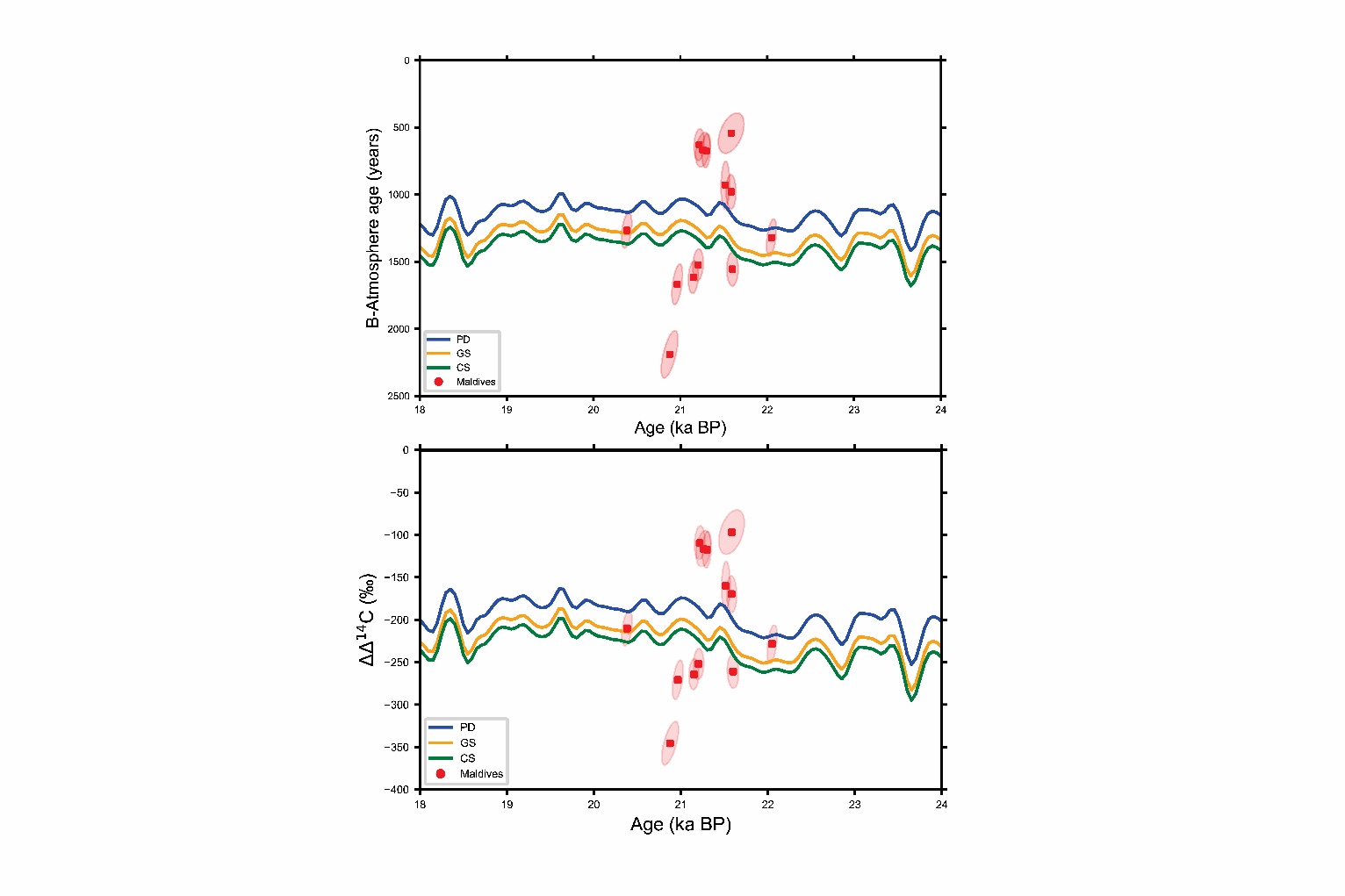


**Figure 2s**: Similar as Figure 5, but here also a comparison of radiocarbon model simulations and the reconstructed and ΔΔ^14^C values (as well as again the B_atm_ ages) are shown. Model simulations are depicted in blue for PD (present control), yellow for GS (glacial ocean) and green for CS (glacial stadial).


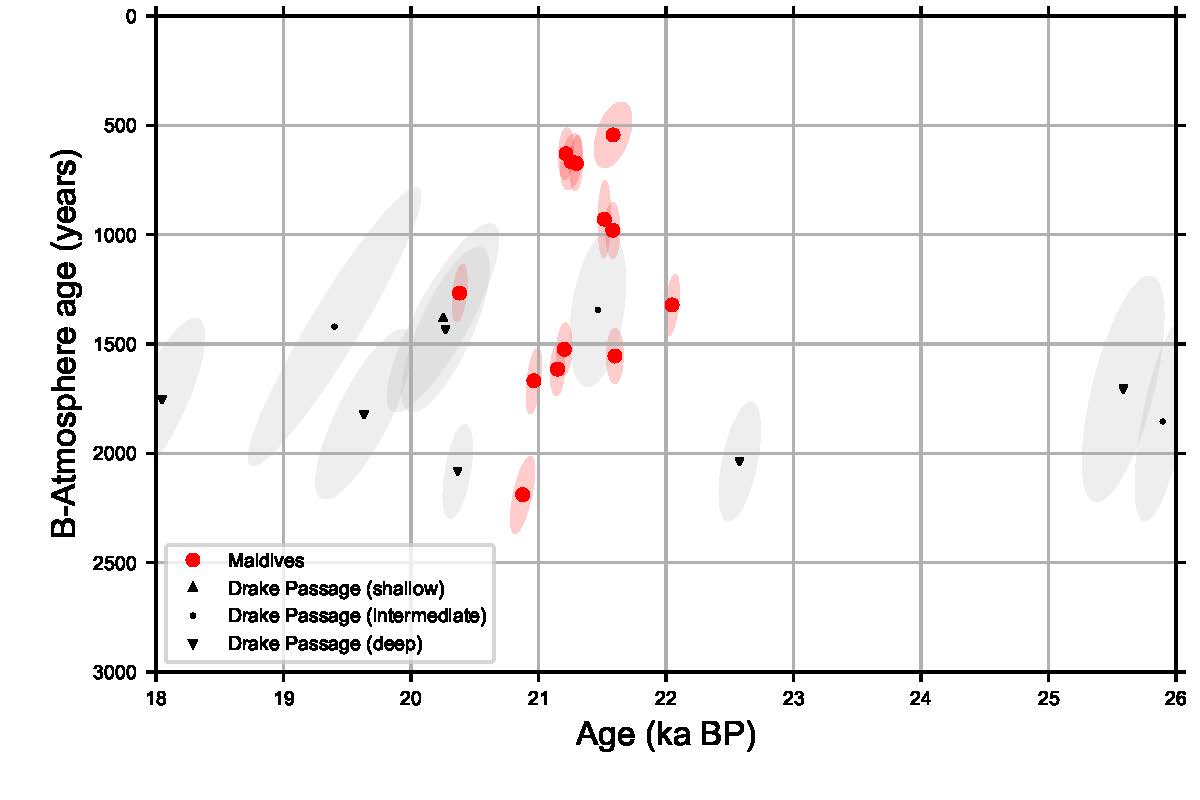


**Figure 3s:** Same as figure 4, but in comparison to B_atm_ ages of different water depth from the Drake Passage^10^.
