# Supplementary material for: Variable ventilation ages in the equatorial Indian Ocean thermocline during the LGM
Source: Sci Rep. 2023 Jul 13;13:11355. doi: 10.1038/s41598-023-38388-z (PMC10345116; doi:10.1038/s41598-023-38388-z)
Supplement: Supplementary file 2 — Supplementary Table 1. [file 41598_2023_38388_MOESM2_ESM.docx]

**Table 1S: Lab and samples Id, Elemental concentration isotopic composition and corresponding ages of the measured cold-water corals species, including sampling water depth**

| **IUP- Code** |  | **sample ID** | **Species** | **Lat** | **Long.** | **238U (µg/g)** | **±** | **232Th (ng/g)** | **±** | **230Th/238U (act. ratio)** | **±** | **230Th/232Th (act. ratio)** | **±** | **d234U (‰)** | **±** | **age uncorr. (ka)** | **±** | **age corr. (ka)** | **±** | **d234Uini. (‰)** | **±** | **water depth (m)** |
| --- | --- | --- | --- | --- | --- | --- | --- | --- | --- | --- | --- | --- | --- | --- | --- | --- | --- | --- | --- | --- | --- | --- |
| IUPH- | 10919 | SO236_7_1 | D. pertusum | 04°09,07′N | 73°29,28′E | 3,81358 | 0,00017 | 0,58222 | 0,0009 | 0,20315 | 0,0005 | 4058 | 11 | 139,54 | 0,64 | 21,325 | 0,057 | 21,287 | 0,06 | 148,18 | 0,68 | 443 |
| IUPH- | 10920 | SO236_7_2 | D. pertusum | 04°09,07′N | 73°29,28′E | 3,49168 | 0,00013 | 0,6079 | 0,001 | 0,20368 | 0,0004 | 3570,5 | 9,1 | 138,35 | 0,49 | 21,412 | 0,047 | 21,368 | 0,051 | 146,95 | 0,52 | 443 |
| IUPH- | 10921 | SO236_7_3 | D. pertusum | 04°09,07′N | 73°29,28′E | 3,2878 | 0,00014 | 3,364 | 0,0063 | 0,2081 | 0,0006 | 621 | 2,2 | 138,98 | 0,5 | 21,911 | 0,071 | 21,66 | 0,15 | 147,74 | 0,54 | 443 |
| IUPH- | 10922 | SO236_7_4 | D. pertusum | 04°09,07′N | 73°29,28′E | 3,60375 | 0,00014 | 1,0764 | 0,0019 | 0,20356 | 0,0006 | 2081,7 | 7,4 | 138,06 | 0,81 | 21,404 | 0,075 | 21,329 | 0,084 | 146,63 | 0,86 | 443 |
| IUPH- | 10923 | SO236_7_5 | D. pertusum | 04°09,07′N | 73°29,28′E | 3,04233 | 0,00014 | 0,6414 | 0,0013 | 0,1966 | 0,0005 | 2848,6 | 8,7 | 142,83 | 0,63 | 20,504 | 0,052 | 20,452 | 0,06 | 151,32 | 0,67 | 443 |
| IUPH- | 10924 | SO236_7_6 | E. rostrata | 04°09,07′N | 73°29,28′E | 4,2926 | 0,00018 | 0,64239 | 0,0008 | 0,2048 | 0,0004 | 4184,1 | 9,4 | 134,46 | 0,58 | 21,625 | 0,046 | 21,587 | 0,049 | 142,9 | 0,62 | 443 |
| IUPH- | 10925 | SO236_7_7 | M. oculata | 04°09,07′N | 73°29,28′E | 4,28171 | 0,00016 | 0,25144 | 0,0005 | 0,20516 | 0,0005 | 10700 | 34 | 134,35 | 0,48 | 21,669 | 0,056 | 21,654 | 0,057 | 142,82 | 0,51 | 443 |
| IUPH- | 10926 | SO236_17_1 | D. pertusum | 04°51.26′N | 73°28.05′E | 2,85643 | 0,00011 | 0,13591 | 0,0004 | 0,19984 | 0,0005 | 12769 | 49 | 134,59 | 0,88 | 21,047 | 0,062 | 21,035 | 0,062 | 142,82 | 0,94 | 455 |
| IUPH- | 10928 | SO236_17_3 | D. pertusum | 04°51.26′N | 73°28.05′E | 3,78334 | 0,00015 | 2,1369 | 0,0045 | 0,2003 | 0,0006 | 1081,2 | 3,8 | 135,15 | 0,57 | 21,088 | 0,065 | 20,947 | 0,097 | 143,38 | 0,61 | 455 |
| IUPH- | 11199 | SO 236 17-6 | D. pertusum | 04°51.26′N | 73°28.05′E | 2,99562 | 0,0001 | 0,4368 | 0,001 | 0,20554 | 0,0006 | 4307 | 15 | 134,67 | 0,63 | 21,707 | 0,065 | 21,67 | 0,066 | 143,16 | 0,67 | 455 |
| IUPH- | 11200 | SO 236 17-4 | M.oculata | 04°51.26′N | 73°28.05′E | 4,02233 | 0,00013 | 0,06878 | 0,0003 | 0,20859 | 0,0005 | 37408 | 183 | 131,89 | 0,56 | 22,123 | 0,057 | 22,119 | 0,057 | 140,39 | 0,6 | 455 |
| IUPH- | 11201 | SO 236 17-7 | D. pertusum | 04°51.26′N | 73°28.05′E | 3,95421 | 0,00009 | 0,35375 | 0,0008 | 0,2012 | 0,0005 | 6898 | 23 | 132,75 | 0,38 | 21,243 | 0,056 | 21,22 | 0,058 | 140,94 | 0,41 | 455 |
| IUPH- | 11202 | SO 236 17-9 | D.pertusum | 04°51.26′N | 73°28.05′E | 2,96618 | 0,00012 | 0,27358 | 0,0006 | 0,20187 | 0,0005 | 6705 | 22 | 133,86 | 0,61 | 21,297 | 0,058 | 21,274 | 0,06 | 142,15 | 0,65 | 455 |
|  |  |  |  |  |  |  |  |  |  |  |  |  |  |  |  |  |  |  |  |  |  |  |
| Comments: |  |  |  |  |  |  |  |  |  |  |  |  |  |  |  |  |  |  |  |  |  |  |
| Age correction according to 232Th concentration and a seawater 230Th/232Th ratio of 8±4. | | | | | | |  |  |  |  |  |  |  |  |  |  |  |  |  |  |  |  |
| Laboratory code/label IUPH: Institute for Environmental Physics, University of Heidelberg, Germany. | | | | | | | |  |  |  |  |  |  |  |  |  |  |  |  |  |  |  |
| d234/238U: measured 234U/238U activity ratios (d234U) are presented as deviation in permil from the equilibrium value. | | | | | | | | | |  |  |  |  |  |  |  |  |  |  |  |  |  |
| d234/238Uini.: Decay corrected 234U/238U activity ratios (d234/238Uini.) are calculated from the given ages; decay constants by Cheng et al. (2013) (URI: https://doi.org/10.1016/j.epsl.2013.04.006). | | | | | | | | | | | | | | | | |  |  |  |  |  |  |
| Decay corrected 234U/238U activity ratios (d234/238Uini.) fits the expected seawater ratio Chutcharavan et al. (2018) | | | | | | | | |  |  |  |  |  |  |  |  |  |  |  |  |  |  |
| Age calculation based on numerically solved equations from Ivanovich and Harmon (1992), age uncertainties are determined using a Monte-Carlo simulation. | | | | | | | | | | | | |  |  |  |  |  |  |  |  |  |  |
