# Supplementary material for: Variable ventilation ages in the equatorial Indian Ocean thermocline during the LGM
Source: Sci Rep. 2023 Jul 13;13:11355. doi: 10.1038/s41598-023-38388-z (PMC10345116; doi:10.1038/s41598-023-38388-z)
Supplement: Supplementary file 3 — Supplementary Table 2. [file 41598_2023_38388_MOESM3_ESM.docx]

**Table 2s: Different Lab Codes, Th/U ages ^14^C ages and the corresponding ^14^ C calculations including B_atm_ ages.**

| **Lab-Code** | | **^14^C-Lab Code** | | **U/Th age** | | | **^14^C age** | | | **F^14^C** | | | **∆^14^C** | **∆∆^14^C** | **B_atm_ age** |  |  |  |
| --- | --- | --- | --- | --- | --- | --- | --- | --- | --- | --- | --- | --- | --- | --- | --- | --- | --- | --- |
|  |  |  |  | (ka BP) | | | (ka BP) | | |  | | | (‰) | (‰) | (yr) | **Species** | **Sample ID** | **Water depth** |
| IUP- | 10919 | MAMS- | 51114.1.1 | 21,216 | **±** | 0,060 | 18,194 | **±** | 0,055 | 0,1038 | **±** | 0,0007 | 353 | -110 | 630 | D. pertusum | SO236_7_1 | 443 |
| IUP- | 10920 | MAMS- | 51113.1.1 | 21,297 | **±** | 0,051 | 18,264 | **±** | 0,058 | 0,1029 | **±** | 0,0007 | 354 | -117 | 674 | D. pertusum | SO236_7_2 | 443 |
| IUP- | 10921 | MAMS- | 51112.1.1 | 21,585 | **±** | 0,148 | 18,334 | **±** | 0,060 | 0,1020 | **±** | 0,0008 | 389 | -97 | 543 | D. pertusum | SO236_7_3 | 443 |
| IUP- | 10922 | MAMS- | 51111.1.1 | 21,258 | **±** | 0,084 | 18,250 | **±** | 0,057 | 0,1031 | **±** | 0,0007 | 350 | -116 | 667 | D. pertusum | SO236_7_4 | 443 |
| IUP- | 10923 | MAMS- | 51110.1.1 | 20,381 | **±** | 0,060 | 18,112 | **±** | 0,056 | 0,1049 | **±** | 0,0007 | 235 | -210 | 1267 | D. pertusum | SO236_7_5 | 443 |
| IUP- | 10924 |  |  | 21,516 | **±** | 0,049 | 18,729 | **±** | 0,084 |  | **±** |  | 312 | -160 | 929 | E. rostrata | SO236_7_6 | 443 |
|  |  | MAMS- | 51108.1.1 |  |  |  | *18,715* | **±** | *0,060* | *0,0973* | **±** | *0,0007* |  |  |  |  |  |  |
|  |  | MAMS- | 51109.1.1 |  |  |  | *18,743* | **±** | *0,059* | *0,0970* | **±** | *0,0007* |  |  |  |  |  |  |
| IUP- | 10925 | MAMS- | 51107.1.1 | 21,583 | **±** | 0,057 | 18,782 | **±** | 0,059 | 0,0965 | **±** | 0,0007 | 314 | -170 | 980 | M. oculata | SO236_7_7 | 443 |
| IUP- | 10926 | MAMS- | 51106.1.1 | 20,964 | **±** | 0,062 | 19,078 | **±** | 0,061 | 0,0930 | **±** | 0,0007 | 175 | -271 | 1668 | D. pertusum | SO236_17_1 | 455 |
| IUP- | 10928 | MAMS- | 51105.1.1 | 20,876 | **±** | 0,097 | 19,488 | **±** | 0,063 | 0,0884 | **±** | 0,0007 | 105 | -345 | 2189 | D. pertusum | SO236_17_3 | 455 |
| IUP- | 11199 | MAMS- | 52707.1.1 | 21,599 | **±** | 0,066 | 19,357 | **±** | 0,058 | 0,0898 | **±** | 0,0007 | 226 | -261 | 1555 | D. pertusum | SO 236 17-6 | 455 |
| IUP- | 11200 | MAMS- | 52708.1.1 | 22,048 | **±** | 0,057 | 19,412 | **±** | 0,058 | 0,0892 | **±** | 0,0006 | 286 | -229 | 1321 | M.oculata | SO 236 17-4 | 455 |
| IUP- | 11201 | MAMS- | 52709.1.1 | 21,149 | **±** | 0,058 | 19,159 | **±** | 0,056 | 0,0921 | **±** | 0,0006 | 190 | -264 | 1615 | D. pertusum | SO 236 17-7 | 455 |
| IUP- | 11202 | MAMS- | 52710.1.1 | 21,203 | **±** | 0,060 | 19,085 | **±** | 0,056 | 0,0929 | **±** | 0,0006 | 208 | -252 | 1524 | D.pertusum | SO 236 17-9 | 455 |
|  |  |  |  |  |  |  |  |  |  |  |  |  |  |  |  |  |  |  |
| Comments: |  |  |  |  |  |  |  |  |  |  |  |  |  |  |  |  |  |  |
| ∆^14^C, ∆∆^14^C and B_atm_ ages: mean values of ellipses | | | |  |  |  |  |  |  |  |  |  |  |  |  |  |  |  |
| * measured twice to check reproducibility, mean ^14^C age used for calibration | | | | | |  |  |  |  |  |  |  |  |  |  |  |  |  |
